# Determining the need for fertility care and the acceptability and feasibility of administering a fertility awareness tool from the user’s perspective in a sample of Sudanese infertility patients

**DOI:** 10.1016/j.rbms.2021.06.004

**Published:** 2021-07-03

**Authors:** R.R. Bayoumi, E. Koert, S. Van der Poel, J. Boivin

**Affiliations:** a Harvard TH Chan School of Public Health, Boston, MA, USA; b Department of Educational and Counselling Psychology and Special Education, University of British Columbia, Vancouver, BC, Canada; c Route de la Capite, Geneva, Switzerland; d School of Psychology, Cardiff University, UK

**Keywords:** cultural appropriateness, FertiSTAT, infertility, preventable causes, qualitative methods, Sudan

## Abstract

Fertility experts have advocated addressing preventable causes of infertility and early intervention. However, awareness of risk factors is low, especially in low- and middle-income countries where the prevalence of infertility is high. To address this lack of awareness, the Fertility Awareness Tool (FertiSTAT) was adapted for use in Sudan and other low-resource countries. The aims of this study were to ascertain the need for fertility education in Sudan (Aim 1), and to gauge the acceptability and feasibility of implementing the FertiSTAT in Sudan (Aim 2), both from the patients’ perspective. Convenience sampling was used to recruit participants for semi-structured-in-depth interviews from a fertility clinic in Sudan. We collected sociodemographic information, medical and reproductive history, asked about fertility knowledge, administered the FertiSTAT and asked about the acceptability of the FertiSTAT. Thematic analysis was conducted for qualitative data. Twenty participants were included; of these, 17 were female, 13 were educated beyond secondary school, the mean age was 32.8 years, and the mean duration of infertility was 4.1 years. Ten themes emerged: of these, three themes addressed Aim 1: ‘desire for fertility information’, ‘state of fertility knowledge’ and ‘benefits of fertility education’; and seven themes addressed Aim 2: ‘specific suggestions for the tool’, ‘factors influencing the acceptability and feasibility of implementing the tool’, ‘challenges and barriers to implementation’, ‘self-disclosure’, ‘understanding of being at risk’, ‘compatibility with worldview’ and ‘cultural tailoring’. Fertility education was viewed as necessary and beneficial; however, participants thought that lack of acceptability of sensitive topics would hinder the implementation of the FertiSTAT. Acceptability and feasibility would be enhanced if challenges were addressed in a culturally sensitive manner using cultural tailoring of materials to increase compatibility with individual worldviews.

## Introduction

Addressing the preventable causes of infertility and early detection of fertility problems through the promotion of fertility care has long been advocated by the World Health Organization (Mascarenhas et al., 2012, Van der Poel, 2012), as well as researchers and clinicians globally (Macaluso et al., 2010; Zegers-Hochschild et al., 2017). Tools to enhance fertility awareness have been developed and used in high-income countries, but inequities remain, especially in low- and middle-income countries (LMICs) where the prevalence of infertility is highest. Primary infertility was reported to be as low as 3.5% in some high-income countries and as high as 9.3% in lower-income countries (Boivin et al., 2007), while secondary infertility was <6% in the USA, >9% in some Middle Eastern countries and up to 16% in sub-Saharan Africa (Mascarenhas et al., 2012, Van der Poel, 2012). In an effort to help bridge this gap, we began the process of adapting a fertility awareness tool (FertiSTAT) for use in LMICs (Bayoumi et al., 2018). The tool was designed in the UK to help women make informed decisions about risks and when to seek medical advice if desired. The FertiSTAT is a 22-item self-administered tool used to increase personal awareness of risk factors that have a negative effect on achieving pregnancy (Bunting and Boivin, 2010). The tool includes lifestyle and reproductive questions, and generates a risk profile with personalized fertility guidance (e.g. to change behaviour). However, to date, the tool has not been adapted for use outside of the context where it was developed.

Sudan is an LMIC with a population of >40 million. Although primary education (age 6–13 years) is compulsory, half of the population remains illiterate (UNESCO, 2017). Estimates of infertility vary from 3% in demographic data (Larsen, 2000) to 80% in clinical studies (Abdalla, 2011, Osman, 2011). More recent studies suggest that primary infertility remains high, at 60% in women attending a fertility clinic (Hussein et al., 2019) and 69% of couples attending a fertility clinic (Elhussein et al., 2019). Infertility has become a concern in Sudanese society as reflected by the growing number of private treatment clinics. However, shortcomings remain in the type and quality of services available, such as minimal specialized training and limited privacy (Khalifa and Ahmed 2012). Research highlights that there is poor fertility knowledge in Sudan (Al Safi, 2007), consistent with the wider (Ali et al., 2011, Dyer, 2008). According to Al Safi (2007), strong gender norms regarding reproductive health place the blame of infertility and the burden of help-seeking on women, who also bear the social stigma of childlessness and are obligated to accept divorce or polygamy as a result of infertility. Conventional knowledge in Sudan assumes that male virility and sexual ability are indicators of fertility; the male contribution to infertility is rarely addressed, and male competence should not be questioned (Al Safi, 2007). This emphasizes the importance of increasing knowledge and awareness of infertility risk factors in Sudan and other LMICs that share similar gender norms.

During adaptation of the FertiSTAT, it was recognized that it would not only need to be translated from English into Arabic, but would also need to undergo cross-cultural adaptation (Bayoumi et al., 2018) to ensure cultural appropriateness (Betsch et al., 2016, Kreuter et al., 2003). Culturally adapted interventions have been associated with better outcomes than standard interventions (Healey et al., 2017). However, as the experiences of people in Sudan with fertility education tools had not been documented, a cross-cultural adaptation process was designed that would facilitate the identification of necessary modifications to address cultural sensitivity and appropriateness, informed by an understanding of the deep structures of Sudanese culture. This process began with an investigation of the comprehensiveness of the risk factors in the FertiSTAT, and a systematic review of identified risks (Bayoumi et al., in preparation). Next, the views of multiple stakeholders about the cultural acceptability and feasibility of implementing the tool in the region were sought (Bayoumi et al., 2018). Qualitative methodology was used in the current study to include the perspective of potential users.

To elicit the points of view of the potential users, in the current study, semi-structured interviews were conducted with Sudanese men and women experiencing fertility problems and attending a semi-private fertility clinic in Khartoum. Aim 1 of this study was to elicit views on the need for fertility education in Sudan, and Aim 2 was to assess the acceptability and feasibility of implementing the adapted FertiSTAT among infertile Sudanese people. Tackling these broad aims allowed consideration of the specific queries and recommendations of stakeholders reported in previous studies, including topics perceived as taboo (e.g. alcohol, drugs and sex with multiple partners) and issues of cultural targeting and tailoring to make the FertiSTAT more culturally sensitive. Cultural targeting is the adaptation of materials to fit target populations, whereas cultural tailoring is aimed at each individual within the target population and is based on characteristics or attributes of that specific person (Kreuter and Skinner, 2000). The present study used interviews as opposed to focus groups because interviews can allow the researcher to gauge the level of difficulty discussing these sensitive topics and reduce social desirability bias, whereas focus groups can intensify self-disclosure (providing information about oneself) issues (Krumpal, 2013).

## Materials and methods

### Participants and recruitment

Convenience sampling was used to recruit male and female patients attending the clinic from January to March 2017. There were no exclusion criteria. Of the 22 patients approached in the waiting room, 20 (91%) agreed to participate and completed the study. Recruitment continued until data saturation was reached, and there was data replication and redundancy, indicating that the point of diminishing returns had been reached (Bowen, 2008).

Ethical approval according to the ethical application processes of both Cardiff University and the University of Khartoum was sought and provided by the School of Psychology, Cardiff University (EC.15.04.14.4130G and EC.07.05.01.1284GR3A7), and by the Department of Obstetrics and Gynaecology, University of Khartoum.

### Materials

The materials used in this study include: the consent form (including briefing); the 16-item background information form; the Arabic FertiSTAT; and the interview topic guide and debriefing (see online supplementary material). The 16-item background information form was used to ascertain demographic, medical and reproductive history (e.g. age, past fertility history). The adapted Arabic FertiSTAT was used to elicit understanding of fertility health issues, and the interview topic guide contained questions about the FertiSTAT.

### Procedure

Patients, who typically wait for several hours (each visit) in the clinic waiting room, were approached and invited to participate in the study. Interested participants were briefed and signed the informed consent form. Participants were allocated an identification number. Research assistants collected demographic data, and RRB conducted the interviews that lasted approximately 30–60 min. During the interview, participants were asked about their fertility knowledge (signs, symptoms and preventable causes of fertility problems), whether they knew when to seek help, and their desire for fertility information, followed by administration of the Arabic FertiSTAT. After completing the FertiSTAT, participants were asked about the acceptability of the tool (topics, potential format, setting, source and timing of administration), and their perception of the potential drawbacks, benefits and utility of the tool. At the end of the interview, participants were thanked for their participation and debriefed. RRB audio recorded and transcribed the interviews.

### Translation

RRB in collaboration with local fertility experts in Sudan translated the adapted FertiSTAT, materials, interview transcripts and illustrative quotes into Arabic. A bilingual Arabic–English linguist from a UK-based translation company (Business Language Solutions) verified the initial translation of the FertiSTAT. Back-translation of random sections of the transcripts/tools (as a sample) was conducted by an independent research assistant for the purpose of quality assurance.

### Research team and reflexive statement

The research team comprised two experienced qualitative researchers. Both researchers were psychologists with content expertise in infertility. RRB was familiar with the cultural context with Sudanese heritage and experience of living in the country. She also had personal experience of the issues related to fertility. This familiarity brought insights to the data from the perspective of an insider. The second researcher, EK, has been working in the field of infertility for the last 10 years. As such, she also brings insights about the phenomena in different populations. Additionally, the fact that she is not Sudanese gave her an outsider view of the culture. This juxtaposition of different positions led to rich discussions during coding and thematic analysis.

### Data analysis

RRB and EK conducted thematic analysis (Braun and Clarke, 2006). Using inductive coding, each coder derived initial codes from the interview data for half of the participants, and discussed the meaning of codes with the other coder through analytic process memos. Disagreements were resolved through discussions until consensus was reached. Coders discussed preliminary thematic groupings of codes to deepen the analytic process to ensure the cohesiveness of each theme and consistency with the overall meanings in the dataset. Coders documented the thematic analysis process, including analytic process memos and reflective notes creating an audit trail. To ensure the trustworthiness of the findings, data collection and analysis was guided by best practice guidelines for qualitative research in the Critical Appraisal Skills Program (Critical Appraisal Skills Program, 2017) and Meyrick (2006).

## Results

### Demographics

Of the 22 patients approached, one declined to participate and one asked to be excused and did not return to complete the study after being briefed about the study (both women). Of the 20 patients who completed the study, 17 were women and three were men. The majority (*n* = 13) were educated beyond high school. The average age of the sample was 32.8 [standard deviation (SD) 9.26, range 22–62] years, the average duration of marriage was 4.9 (SD 3.58) years, and the average duration of infertility was 4.1 (SD 2.88) years. The self-reported reason for infertility was classified as ‘female factor only’ in 12 cases (60%), ‘male factor only’ in two cases (10%), and ‘both male and female factor’ in three cases (15%). The reason was ‘unknown’ in one case (5%) and ‘still not diagnosed’ in two cases (10%). Five (25%) women reported previous pregnancies, but only two (10%) had live births, and one (5%) woman was currently pregnant (first trimester) following treatment.

### Results of thematic analysis

Thematic analysis resulted in 10 themes addressing the two aims of the study. Three themes that addressed Aim 1 (need for fertility education in Sudan) emerged from the thematic analytic process, and seven themes addressed Aim 2 (acceptability and feasibility of implementing the FertiSTAT in Sudan). Table 1 shows the themes and illustrative quotes. Although some of the themes overlapped, they were not grouped together (to illustrate how they emerged from the data); instead, the redundancy was eliminated during the integration of themes to generate the thematic map.

**Table 1:** Themes that emerged from thematic analysis of patient interviews in Sudan.

	Themes	Description of theme	Illustrative quotes
A	Themes addressing Aim 1		
1	Desire for fertility information	Fertility information that was desired and if it was generated or endorsed	ID5: ‘yes, I’m currently searching [for information]’
2	State of fertility knowledge in this sample • What is known • What is not known • Misconceptions/myths	Current fertility knowledge, gaps in knowledge, and misconceptions or myths about fertility	ID20: ‘yes after 35 the chance is weak, very weak’ ID13: ‘I would say a year is good’ ID1: ‘I feel I have little information’ ID17: ‘no I didn’t know, especially the specific age I didn’t know that’ ID13: ‘cleanliness and things like that’ [referring to causes of infertility]’ ID16: ‘[ovarian] cysts always’ [referring to causes of infertility]
3	Benefits of fertility education • Perceived personal benefit (to self) • Perceived general benefit (to others) • Utility of the tool: addresses knowledge gap and encourages behaviour change	Potential benefits of implementation of the tool to the participants (self), to people in Sudan generally (other), and the potential uses of the tool	ID2: ‘yes I would look at it, I would find it beneficial’ ID17: ‘our society is in need of lots of raised awareness, A LOT!!’ ID13: ‘to see where there are gaps and to fill them’
B	Themes addressing Aim 2		Illustrative quotes
1	Specific suggestions for the tool • Content: taboo topics • Format: print versus seminar • Setting: schools, home etc. • Source: doctor, specialist etc. • Timing: puberty, before marriage etc.	Specific comments/suggestions about aspects of the tool and its implementation	ID19: ‘maybe in the olden days maybe, but now it’s ‘aadee’ [normal] (…) if you introduce yourself properly in the beginning and they see you are a doctor, a professional [then they would be more willing to accept these taboo topics]’ ID19: ‘something printed the boys will not read it (…) if its lectures or seminars (…) they will accept it, they will listen, because a boy by nature wants to hear not to read’ ID13: ‘I imagine the home to be the best context, I mean the most important role, one sees their father and their mother and how they are, it’s better that they show them’ ID10: ‘your mother, older sister at home’ ID19: ‘the real difference lies in whether the information was given by a specialist, not man or woman’ ID12: ‘it could be specifically for women, a seminar just for women so they can ask’ ID18: ‘I think at puberty they should be made aware of these things’ ID19: ‘when they are in the engagement period, approaching marriage’ ID16: ‘from early on is better so I can avoid things like drinking too much coffee and tea and things like that’ ID15: ‘every girl MUST go and get checked out before she gets married’
2	Factors influencing implementation • Endorsed: – Personal preferences – Perceived benefit • Participant generated: – Acknowledging the benefit of education/information – Appropriate method of distribution – Persistence	Factors affecting tool implementation endorsed by the participantsFactors affecting implementation generated by the participants	ID1: ‘it’s choices, you don’t like the page, you turn it’ ID1: ‘clear and direct questions so that the answer is clear and direct, you benefit and I benefit’ ID14: ‘if it [FertiSTAT] is distributed right’ RRB: so, you’re saying even if they say they don’t accept it, we should give it anyway? ID14: ‘I told you, he will calculate it [risk level] in his head. He might think maybe this is right, he will do it himself [fill out FertiSTAT]’
3	Challenges and barriers to implementation • Others will not accept taboo topics • Openness to health education in general and fertility specifically • Implementation may be dependent on level of understanding, knowledge, education and religiosity • Source not trusted	Challenges and barriers to successful implementation of the tool	When asked if she would accept the materials: ID5: ‘yes acceptable’ [but when asked if others would accept it, her response was different] ‘some people will consider it and others will not’ ID4: ‘people may not accept these subjects’ ID1: ‘cons, there are no cons for me, the topic is normal’ RRB: ‘do you think people will respond authentically?’ ID1: ‘no (…) from the beginning, you will get a sense of whether this person is willing to accept things, or not accept, for example, this sex question, most people will say “enough I don’t want to (continue)”’ ID11: ‘(…) you will face difficulties, you will face unacceptance of the idea itself. I’ve done village work, acceptance of things like this was problematic for people. To communicate to them about family planning and to prevent circumcision of females and things like that, we faced problems, our problem is our customs’ ID14: ‘it will depend on their level of understanding, they may not accept it. Not everyone will accept, everyone has a different level of understanding’ ID1: ‘the religious one, in a religious way (…) God has forbidden certain things because they (the forbidden actions) can harm us’ ID13: ‘it seems that it’s always the case that if you trust the source [person] that the information is coming from, that’s better. But if it comes from someone I don’t trust, I will just leave him and go’
4	Self-disclosure	Factors that affect self-disclosure, e.g. social norms, social desirability, demographics.How issues of self-disclosure were resolved internally: self–other as a resolution for internal conflict of modern–traditional, cultures in transition, pull between modern versus traditional values.When is self-disclosure important (practice versus research)	RRB: ‘is there anything else you could add that you think would help us?’ ID1: ‘no, your way is nice’ ID19 (graduate level education): ‘OK you really have to write this [more research on varicocele] in the recommendations!!’ RRB: ‘so it’s not a problem, for example we say ‘this area, people should not talk about’?’ ID8 (62-year-old man): ‘it’s WRONG not to talk about it!!’
5	Understanding of being at risk	Aspects that affect our understanding of being at risk, e.g. demographics, previous knowledge and experience, culture (social norms, religion)	ID7: ‘everyone knows what can harm them and can help them, and they are still doing the [behaviour that is] wrong, like, for example, sex, they know it can transmit diseases but they still do it. They use protection and say ‘I won’t get a disease’. They know everything but they try in different ways to do things, but this thing [premarital sex] is haram [forbidden by Islam] and wrong’ ID1: ‘before marriage (…) I felt like I didn’t want to educate myself’ ID14: ‘They should show this to the men too, so they don’t say it’s just from the woman [the fertility problem], they have to, they have to know it, this thing especially, boys will be boys, so you know boys can have relations [sex] as much as he wants before marriage and stuff, and then he comes and then, I mean after marriage he will have repented to God [no longer engages in sex with anyone other than his wife] and they have no problem [no extramarital affairs]’ RRB: ‘was the information beneficial? And was there any information you were not aware of before?’ ID6: ‘yes, useful, I’ve seen it before’ ID15: ‘every girl MUST go and get checked out before she gets married, to get herself checked, I had problems with my period, and I was not bothered with it’ ID1: ‘God has forbidden certain things because they can harm us’ ID13: ‘So, knowing about this, awareness about such things especially here in Sudan, here the girl won’t go to the doctor no matter what. For example, if her period is late she should find out, if her period she could have a problem, go to the doctor’
6	Compatibility with worldview	Compatibility of information with worldviews, social norms, beliefs and values that affect the acceptability and feasibly of using the tool in Sudan and the issues related to self-disclosure and understanding risk	ID5: ‘this is a type of education and [education] is not wrong’ ID1: ‘sex outside marriage is haram [forbidden by Islam], God has forbidden certain things because they can harm us’ ID7: ‘And I tell you something, in this day and age, they all know, they know wrong from right. And they are doing the wrong (regardless). Everyone knows what can harm them and can help them. And they are still doing the wrong, how, like, for example, sex, they know it can transmit diseases but they still do it. They use protection and say ‘I won’t get a disease’. They know everything but they try in different ways to do things, but this thing [premarital sex] is haram [forbidden by Islam] and wrong’ ID13: ‘yes, early is 1 year, some people wait 4 or 5 years to get tested, no I mean you have just wasted time like this. It’s better that they find out, so that even if God did not will it [meaning you can’t have babies], you can separate. Sometimes there are people that God gives them [a baby] with someone else, it was not meant to be here [in the first marriage]’
7	Cultural tailoring	How the tool could be tailored to fit the culture, i.e. according to gender, age, level of education or understanding and religiosity	ID14: ‘(…) printed materials, posters, pamphlets that can reach the mum or the aunt at home, they read it. People who can’t read [illiterate] can get it at the mosque, you give the information to the imam [priest] and tell him to convey. This way the people at the mosque will know something and the mums will get the printed material’ ID1: ‘the religious one, in a religious way, that sex outside marriage is haram [forbidden by Islam], God has forbidden certain things because they can harm us’ ID10: ‘it’s better from a woman of course! (…) a man, for example, I can’t ask him questions, but you are a woman like me so I can ask you questions’ ID1: ‘you reach her at her level of understanding, each person at their level of understanding’

ID, participant identification number; RRB, Rasha R. Bayoumi (interviewer); tool, Fertility Awareness Tool (FertiSTAT).

#### Themes addressing Aim 1

Three themes that addressed Aim 1 of this study (need for fertility education in Sudan) emerged from the data (see Table 1). The first theme was the ‘desire for fertility information’. The data provided evidence of unanimous endorsement of a desire for information about fertility. A few participants also indicated that they were actively looking for information. The second theme was the ‘state of fertility knowledge’ in this sample. Most of the participants seemed to be aware of the impact of age on female fertility, and were aware that after a couple has been trying for a baby for 1 or 2 years without success, they should go to the doctor. However, when participants were asked if they had fertility knowledge, many stated that they did not. There were also misconceptions/myths held by some participants regarding risk factors for infertility and isolating certain factors as the only cause of infertility (see Table 1 for examples). The third theme was the ‘benefits of fertility education’. The benefit to self and others was expressed by most of the participants, and the utility of the tool to addresses knowledge gaps and to encourage behaviour change was noted.

#### Themes addressing Aim 2

Seven themes addressed Aim 2 of this study (acceptability and feasibility of implementing the FertiSTAT in Sudan) (see Table 1). The first theme was ‘specific suggestions for the tool’, which related to the content (FertiSTAT items), best timing for implementation of the FertiSTAT and context of its delivery. When asked about whether they thought the sensitive topics would hinder acceptability, one participant stated:

Maybe in the olden days maybe, but now it’s ‘aadee’ (normal) [and she noted that it would depend on how the provider was viewed:]. If you introduce yourself properly in the beginning and they see you are a doctor, a professional [then they would be more willing to accept these taboo topics] (Participant 19).

The best context for implementation of the FertiSTAT referred to format, setting and provider. All participants endorsed a magazine version and some generated format examples including seminars and print materials, although there was disagreement about print materials. The most suitable setting suggested by most participants was educational institutions (schools and universities), although some suggested that the home might be more appropriate. The participants stated that the most suitable source to provide this fertility information was a doctor, a professional or a specialist. These responses demonstrated that the perception of the source as knowledgeable was more important than the source’s profession or gender. Some participants thought that a same-gender source would be better, while some also thought that a family member (eg. a parent or older sibling) should convey this type of information. Most participants stated that the most suitable time for delivery of this information was at an early age – puberty (adolescence) and the engagement period (before marriage) – when it was thought that women can make changes to safeguard their fertility and seek early treatment. For example:

… awareness about such things, especially here in Sudan – here the girl won’t go to the doctor no matter what. For example, if her period is late, she should find out; she could have a problem [and should] go to the doctor (Participant 13).

The second theme concerned ‘factors influencing the acceptability and feasibility of implementing the FertiSTAT in Sudan’. Most participants thought that personal preferences would dictate whether or not the FertiSTAT was acceptable. It was also noted that the perceived benefits of the tool would influence its acceptability, and that acceptability would be improved through appropriate methods of distribution and persistence in providing the information.

The third theme was ‘challenges and barriers to implementation’. Four potential barriers or challenges to implementing the FertiSTAT in Sudan emerged. Most participants stated that they would find it acceptable to talk about taboo topics but that others would not. When asked if she would accept the materials, one woman said:

Yes acceptable [but when asked if others would accept it, her response was different]. Some people will consider it and others will not (Participant 5).

The lack of openness about health education in general and fertility specifically was expressed as a possible barrier. Implementation was viewed as dependent on the levels of understanding, knowledge, education and religiosity. Finally, the participants thought that the trustworthiness of the source could be a potential challenge.

The fourth theme was ‘self-disclosure’. Evidence for this theme came from several observations. First, participants were unwilling to self-disclose about behaviours that were against social norms in Sudan (e.g. none of the 17 women reported sexual activity before marriage). Second, agreeableness, wanting to be sociable or aiming to please others, emerged because most participants just endorsed the FertiSTAT as it was. Third, the participants who felt able to self-disclose (i.e. not affected by social desirability or agreeableness) were those who would be allowed to violate norms in Sudanese society, namely those perceived as ‘higher up’ in the social hierarchy (men, older people, more educated people).

The fifth theme was ‘understanding of being at risk’, that is, the individual’s perception or thinking about health risks in general (what causes ill health) and as applied to infertility (what causes infertility, who is at risk, what types of behaviours increase risk). Information in the data and in general led to the identification of several factors that affected ‘understanding of being at risk’. One participant’s understanding seemed to be informed by a combination of religious doctrine and previous knowledge of disease transmission. It appeared that the understanding of being at risk could differ by age; for example, younger people appeared to feel more invincible. There also appeared to be gender norms about behaviour and risk taking:

They should show this to the men too, so they don’t say it’s just from the woman [the fertility problem], they have to, they have to know it, this thing especially, boys will be boys, so you know boys can have relations [sex] as much as he wants before marriage and stuff (Participant 14).

This quote reflected the participants’ general understanding that although social norms allow premarital sex for men, this still may be a risk; thus, the understanding of being at risk was partially based on gender. The data demonstrated that understanding of being at risk could be informed by previous knowledge, information, and personal experience of infertility specifically or medical issues more generally. Another participant expressed that had she known about the signs of fertility problems such as irregular periods, she would have sought treatment earlier. Understanding of being at risk was also informed by what is forbidden by social norms, laws or religious doctrine, and can affect behaviour.

The sixth theme was ‘compatibility with worldview’. The participants’ responses demonstrated that if health information/education was perceived to be compatible with a personal worldview (values, beliefs, philosophy), it was more likely to be taken up and assimilated. When information was not congruent with personal worldviews, it could be disregarded or discredited. For example, several participants expressed the general Muslim society belief about the value of knowledge: ‘this is a type of education and (education) is not wrong’ (Participant 5). Participants stated that Islam forbids some of the risk factors for infertility identified in the FertiSTAT. One female participant explained, ‘God has forbidden certain things because they can harm us’ (Participant 1).

The seventh and final theme was ‘cultural tailoring’. The main idea of this theme was that tailoring health messages of an educational tool like the FertiSTAT, to make them more compatible with the user’s worldview, would make the tool more acceptable. For example, a female participant said:

… printed materials, posters, pamphlets that can reach the mom or the aunt at home, they read it. People who can’t read can get it at the mosque, you give the information to the imam [priest] and tell him to convey. This way the people at the mosque will know something and the mums will get the printed material (Participant 14).

Tailoring was suggested according to three factors: religiosity, education or level of understanding, and gender.

**Fig. 1:**
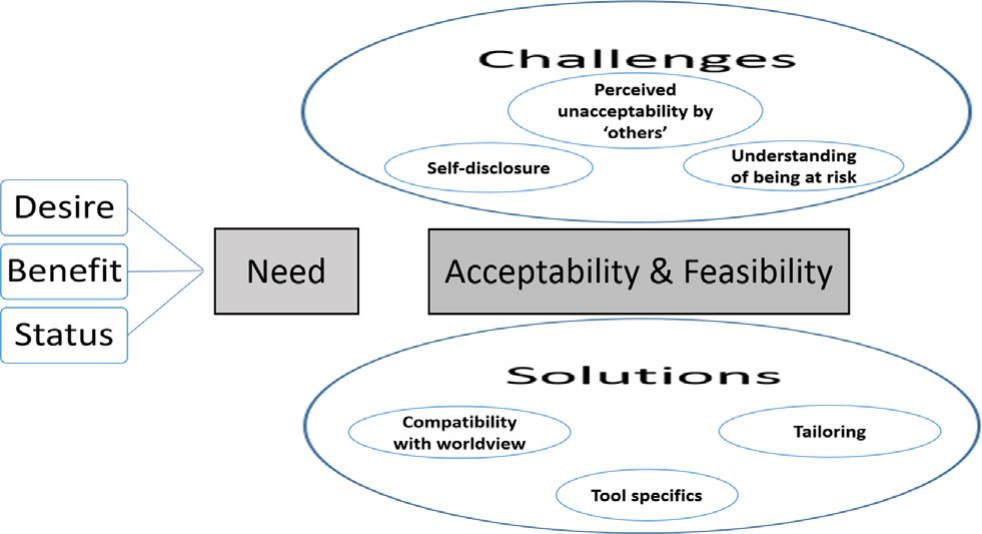
Map of themes and how they apply to the aims of the study. This figure demonstrates the interconnectedness of the themes that emerged from the data and how they relate to the study aims. In the figure, ‘Need’, referring to the need for fertility education in Sudan (Aim 1), was informed by perceived desire for and benefit of fertility education, and the state of fertility knowledge. Aim 2 [the acceptability and feasibility of implementation of the Fertility Awareness Tool (FertiSTAT) in Sudan] was affected by the challenges and solutions. Potential challenges included: factors that lead to an understanding of being at risk, factors influencing self-disclose of sensitive information and perceived acceptability of FertiSTAT by other people. Potential solutions included: specific changes to FertiSTAT, compatibility of FertiSTAT with an individual’s worldview, and cultural tailoring of FertiSTAT to be congruent with an individual’s worldview.

The coders’ integration of themes with the aims of the study led to the development of the map depicted in Fig. 1. ‘Desire for more information’, ‘benefit of fertility education’ and the ‘state of fertility knowledge’ appear to inform the need for fertility education (Aim 1). The other themes were perceived to be either challenges or solutions regarding the acceptability and feasibility of using the FertiSTAT in Sudan (Aim 2).

## Discussion

### Principal findings

These findings indicated that fertility education was perceived to be necessary and beneficial, and that the FertiSTAT would be acceptable, and its implementation would be feasible only if challenges were addressed in a culturally sensitive manner. These challenges included the difficulty of accepting communication about sensitive topics, issues of self-disclosure, and understanding of being at risk. Approaches to address these challenges included changes to the format of delivery (e.g. all-women seminars) that would reduce the impact of social hierarchy and could facilitate self-disclosure. In addition, it was inferred that cultural tailoring to make materials compatible with individual worldviews was a solution to generate a personalized understanding of being at risk, and to enhance acceptability of information about sensitive topics. An example of cultural tailoring of the FertiSTAT would be to have provider-administered versions of the FertiSTAT (checklist, flipchart) to enable cultural tailoring of information to each user’s level of literacy and cultural attributes. The materials would have instructions for the provider to gauge the level of education and/or religiosity of each user, and to discuss the materials in the way that is most suitable to each specific user based on that information.

Results showed a need for fertility education stemming from a lack of fertility knowledge among people in Sudan that was comparable with reports in the literature from developed (Bunting et al., 2013) and developing (Ali et al., 2011, Ola et al., 2010) countries. It was inferred from the data that the tool as presented would only be acceptable and feasible if it was compatible with the Sudanese culture. This was consistent with studies emphasizing that successful implementation of health promotion in culturally diverse settings hinges on achieving accurate cultural sensitivity in health messaging (Betsch et al., 2016, Kreuter et al., 2003, Resnicow et al., 1999). Beyond cultural targeting, it was also inferred that the materials needed to be compatible with each user’s specific level of cultural attributes. The present findings suggest that one way to achieve this compatibility, ascertained from the results, was to tailor the materials with an understanding of the deep structure of the society to fit each user’s specific level of sociocultural factors, such as religiosity and education (Kreuter et al., 2003, Resnicow et al., 1999). Some challenges were identified and specific changes to the tool were suggested to tackle these challenges.

#### Challenges to implementation

There were three main challenges to implementing the FertiSTAT in Sudan ascertained from the data. One challenge was that although all the participants expressed that they accepted the materials for themselves, some felt that other people in Sudan might not accept the tool. This self-versus-other dichotomy could reflect several processes. First, it could be that people were not willing to openly disclose their own views of the tool, and projected their objections on to others. This is not surprising given that being agreeable, cooperative and helpful is valued in Sudan. Second, this dichotomy could be a manifestation of the pull between modern and traditional values inherent in cultures in transition, as is the case for Sudan. Participants most often highlighted the self–other dichotomy when considering the acceptability of addressing taboo topics in the tool. In this way, the participants could convey a modern view of self while projecting the negative ‘traditional’ beliefs on to others as a way of maintaining aspects of both tradition and modernity within one’s persona. Therefore, it appeared that this sample may be liberal and willing to engage in premarital sex (more modern), yet still feel hampered in disclosing sexual history in the interview due to fear of being judged according to traditional norms forbidding premarital sex (laws and religious doctrine). These findings highlighted the need to address such dichotomies when tailoring health promotion tools to individual preferences and worldviews (Kreuter and Skinner, 2000).

The second challenge was willingness to self-disclose less favourable aspects of the self. Results suggested that Sudanese users might not be as willing to self-disclose as they should be about their lack of knowledge or exposure to particular risks (which would be to their benefit), as noted previously in the literature (Gerbert et al., 1999, Krumpal, 2013). This unwillingness to disclose seemed to be mainly due to worry about creating bad impressions with others (spouse, doctor). The consequence of lack of self-disclosure in clinical contexts is critical because it can lead to less accurate diagnosis and management (Gerbert et al., 1999). For example, not being forthcoming about smoking or alcohol consumption has been noted by fertility doctors as a challenge to accurate diagnosis of a fertility problem (Shady Grove Fertility, 2016). Self-disclosure can also be viewed as a challenge to health education as it would reduce the provider’s assessment of perceived comprehension by the user (Krumpal, 2013).

A third challenge to risk communication was perceived understanding of being at risk. It has been reported that to avoid hazards such as smoking, people need to understand what it means to be at risk (Weinstein, 1999). This perception of risk is influenced by several factors, including psychological and cultural factors such as attitudes and values (Boholm, 1998, Sjoberg, 2000). It was inferred from the data that understanding of risk was moderated by personal characteristics. The data in the current study highlight the importance of recognizing that personal risk or susceptibility is influenced by several features such as age and gender, but also by social norms and culture (Boholm, 1998, Sjoberg, 2000, Weinstein, 1999). It could be inferred from the data that youngsters are perceived to be uninterested in health education, possibly linked to the idea of lack of perceived risk associated with age. Further, the data suggested that perception of risk might be related to gender. For example, the assumption that infections affect women alone, and therefore sexual behaviour of women alone is important, is consistent with reports in the literature of women perceiving themselves as being at higher risk and men perceiving themselves as being at lower risk (Boholm, 1998, Fiuncane et al., 2000). Several participants emphasized the inclusion of men in fertility education, in line with the importance of addressing men in gender-neutral health education noted in the literature (Östlin et al., 2006). Integration of gender into health programmes (inclusion of gender norms and taking gender-based inequalities into account) has been reported as a way to achieve positive reproductive outcomes (Boerder et al., 2004, Robertson et al., 2008).

Another example highlighting the complexity of understanding risk is the common belief that unmarried girls should not seek treatment by a gynaecologist, even if they are having menstrual problems. This belief results as a combination of two factors. First, the misconception that gynaecologists treat issues related to sexual health alone (e.g. sexually transmitted infections and infertility). Second, the pervasive cultural assumption that unmarried girls are not having premarital sex. The combination of these two ideas would suggest that unmarried girls should not see a gynaecologist; even if there is an understanding that menstrual problems can lead to infertility, it would be culturally more acceptable to go to the gynaecologist after marriage. Thus, the lack of unmarried girls visiting gynaecologists reflects a lack of knowledge about non-sexually-related gynaecological diseases affecting fertility (e.g. anovulation) and a denial about premarital sex in girls. Implementation of the FertiSTAT and provision of reproductive health education infographics by healthcare workers at community level (e.g. in schools and mosques) can provide information about when to seek help that would enable both the girls and their mothers to make informed decisions about if/when to visit a gynaecologist. The wide dissemination of these materials within the community would increase the general level of fertility awareness, and could therefore potentially make gynecological visits before marriage less taboo.

#### Potential solutions to identified challenges

Participants generated multiple suggestions for where and how the tool could be implemented, such as information leaflets or same-sex seminars targeting adolescents and those about to embark on marriage in schools and universities. These results were in line with current recommendations of the Sudanese Federal Ministry of Health that health promotion should focus on community-based interventions, and that schools are a setting where child, parent and teacher involvement could enhance health promotion efforts (Elsubai, 2007). Many participants stated that a doctor would be the ideal source to disseminate the information because they are perceived to be knowledgeable and trustworthy, corresponding with the idea that doctor–patient communication could be viewed as a basis for motivation, reassurance and support (Betsch et al., 2016).

The findings suggested that people might be more willing to accept health-based educational materials that are compatible with their worldview that can then be integrated into one’s understanding of a concept. The participants’ recommendations underscored the need to tailor materials to the individual to be congruent with their abilities and views. Cultural congruency between the message and each user’s cultural attributes has been suggested to enhance effectiveness and lead to better outcomes (Betsch et al., 2016). The fact that the information about congruence and tailoring emerged from the data organically without being sought actively supports the legitimacy of such claims in the literature (Govender, 2005, Kreuter et al., 2003, Timmerman, 2007).

Activities of adaptation of the FertiSTAT reported thus far have followed methods of cultural targeting to address cultural sensitivity. However, the current study underscored that effective implementation of the FertiSTAT needs to go beyond cultural targeting that reflects an understanding of deep structures, to include cultural tailoring based on each individual’s level of cultural attributes to achieve maximum impact.

### Limitations and strengths

Given that this was a very small, convenience sample, mainly consisting of women receiving treatment in a semi-private facility in the capital city, the generalizability of the findings is limited. However, the goal of qualitative research is not generalization, but rather understanding peoples’ perspectives and experiences from their personal stories, and comparing cases to discover patterns and themes (Patton, 2014). Feasibility questions relating to whether the intervention can be used in a new target population, and the most appropriate methods of delivering the intervention can be best answered through qualitative research (Bowen et al., 2009). It is also important to note that recruitment continued until saturation was reached, indicating that the sample size was large enough to fully capture the experience of women in this setting (Bowen, 2008). The representativeness of the sample was another limitation because of the lack of male participants; the average age at marriage was older than the national average in Sudan (United Nations, 2015); the proportion of male factor infertility was smaller than that reported globally (Kumar and Singh, 2015); and the sample was mainly urban and educated. The education level of the sample exceeded that of the national average; therefore, the acceptability and applicability of the FertiSTAT within the Sudanese population in general might be different from the current sample. These limitations necessitate replication with more diverse samples.

The main strength of this study was the adherence to best practice guidelines of qualitative analysis (Braun and Clarke, 2006, Critical Appraisal Skills Programme, 2017, Meyrick, 2006). This included independent coding, double checking and discussion of coding, and thematic analysis with ongoing documentation of the analytic process through an ‘audit trail’. Issues of researcher bias and reflexivity were discussed between coders to enhance the trustworthiness of the findings. Another strength was that RRB was from the target population, which is consistent with ‘constituent-involving approaches’ that suggest that the inclusion of indigenous staff leads to awareness about cultural features that go beyond the obvious observable characteristics such as language (Kreuter et al., 2003).

### Implications of the findings

The findings lend support to the idea that culturally acceptable implementation of health-promoting interventions such as the FertiSTAT require cultural sensitivity and tailoring of tools to the level of understanding and conservativism (modern versus traditional) of the population and the individual. To promote fertility awareness, a safe and more effective way to talk about sex in a conservative society like Sudan would be to address sex within the context of marriage, and with the aim of achieving reproductive goals. This can be achieved through an integrated awareness campaign that includes information about sexual education, contraception and infertility within one comprehensive pamphlet or poster. Such a campaign could be integrated within existing healthcare and referral systems, and could be available at all levels of health care. It could also be disseminated in schools, universities and public places (e.g. markets, mosques).

The need for several versions of the adapted tool noted by the stakeholders (Bayoumi et al., 2018) was confirmed from the data in the current study. Versions need to be specific to the target audience and setting; adolescent boys might not respond to pamphlets but will be interested in seminars, young girls might be more willing to engage with a female provider, and provider-administered versions would allow for tailoring to individual needs. For example, a flipchart modality can be used as an educational tool, a checklist can be used as a screen and as an ‘ice-breaker’ to start communication or as a starting point for discussion between patient and provider, a pamphlet can be used as a way for people to talk about their fertility issues with each other, and a poster can be a way to introduce sex education and contraception within a culturally sensitive and acceptable context. In the adapted flipchart version of the FertiSTAT (Bayoumi et al., 2018), cultural tailoring can be achieved by adding questions on the provider side to help the provider gauge the level of understanding, education, religiosity and conservativism, and tailor the materials to each individual’s specific level. For example, questions that include information of a technical nature (e.g. medical terminology such as ‘endometriosis’) can be explained in a more rudimentary way to users who appear to be less educated. Similarly, if the provider perceives the user to be conservative/religious, s/he can modify questions pertaining to taboo topics such as having sexual intercourse with multiple partners or using alcohol or drugs. In this way, the provider is sharing information in a less blunt manner rather than asking the user to answer these questions directly.

### Future research

Future research requires more focus groups in more countries, different samples (e.g. men, rural), and formal translation and back-translation of tools into Arabic (and other languages, as required). Small-scale roll-out of Arabic versions of tools in several locations (urban and rural) is needed, followed by large-scale countrywide roll-out. Simultaneously, the protocol for cultural adaptation of the FertiSTAT could be replicated in other regions (e.g. Asia). Most importantly, there is a need to conduct follow-up studies and measure the impact after roll-out of the FertiSTAT on outcomes such as behavioural change in lifestyle (e.g. less smoking) and change in help-seeking practices (e.g. visiting the gynaecologist for menstrual dysfunction), as well as changes in guidelines, policies and provider behaviour.

## Conclusion

Tackling the preventable causes of infertility using tools that improve fertility awareness, such as the FertiSTAT, can be realized in LMICs, most critically through the adaptation of these tools by addressing culturally sensitive topics. The importance of culturally sensitive health promotion echoed in the data is captured by the Arabic saying ‘no embarrassment in knowledge’. Successfully implementing the FertiSTAT in Sudan and the Middle East would therefore require integration of cultural targeting and tailoring, and incorporating the specific suggestions (format, setting, source and timing) to address perceived challenges to its effective use. Addressing challenges, such as the transition between modern and traditional societies, issues of self-disclosure and understanding of being at risk, identified through personalized cultural tailoring would be the most effective way to achieve cultural sensitivity through congruence with individual worldviews. Additionally, personalized messaging has the potential to provide benefits similar to those demonstrated regarding personalized patient-centred care in other areas of health. Caution should be exercised when generalizing to populations with low levels of education as this may affect the acceptability and implementation of the FertiSTAT. Finally, the lessons learned extend beyond implementation of the FertiSTAT from fertility awareness to health promotion in general.

## Declaration

*The authors report no financial or commercial conflicts of interest.*
