# Supplementary material for: Determining the need for fertility care and the acceptability and feasibility of administering a fertility awareness tool from the user’s perspective in a sample of Sudanese infertility patients
Source: Reprod Biomed Soc Online. 2021 Jul 3;13:85–97. doi: 10.1016/j.rbms.2021.06.004 (PMC8340046; doi:10.1016/j.rbms.2021.06.004)
Supplement: Supplementary Data 1 [file mmc1.docx]

**Patient interview materials**

A. Procedure

B. Consent form

C. The 16-item background information form

D. Semi-structured interview topic guide

E. Debrief for interviews

1. **Procedure**

Step 1: Briefing and consent form

Step 2: Background information

- Administer the 16-item background information form

Step 3: Questions regarding fertility awareness

- Assess whether the participant knows about the following:
  - Signs and symptoms of fertility problems
  - Preventable causes of fertility problems
  - When to seek help if they have trouble becoming pregnant

Step 4: Desire to know about fertility

- Ask if the participant would value knowing more about these aspects of fertility?

Step 5: Administration of FertiSTAT

- Administer the adapted FertiSTAT checklist (Arabic version)

Step 6: Questions about FertiSTAT

- Ask open-ended questions to assess the following:
  - How they talk about fertility health to others and what is their preferred language of communication for sensitive topics
  - Potential format of administration (e.g. specific format, setting, source and time required for administration
  - Would they have wanted to know this information in the past?
  - How useful would it have been to have this information and when?

Step 7: Debrief

1. **Consent form for FertiSTAT interviews**

**Consent form** (to be read or read out)

I understand that my participation in this project will involve a ‘think-aloud’ task during which I will provide my thoughts and feelings about the FertiSTAT while completing the tool. I will then be asked to complete a short interview and a questionnaire about my demographics (e.g., age, education). The whole study should take around 60 to 90 minutes to complete.

I understand that participation in this study is entirely voluntary and that I can withdraw from the study at any time without giving a reason.

I understand that I am free to ask any questions at any time. I am free to withdraw or discuss my concerns with the researcher Rasha Bayoumi (bayoumir@cardiff.ac.uk).

I understand that the think-aloud task and interview will be audio-recorded so that the topics raised can be transcribed and synthesised. The information provided by me will be stored on a password-protected computer that belongs to the researcher Rasha Bayoumi and Prof Jacky Boivin and will be held anonymously, so that it is impossible to trace this information back to me individually. Once the recording of the session has been transcribed the recording will be deleted and the transcribed data and questionnaire responses will be retained indefinitely.

I also understand that at the end of the study I will be provided with additional information and feedback about the purpose of the study.

I, ___________________________________(NAME) consent to participate in the study conducted by Rasha Bayoumi, School of Psychology, Cardiff University with the supervision of Professor Jacky Boivin.

Signed:

Date:

**Arabic Consent form**

استمارة الموافقة (أن تقرأ أو تقرأ)

أنا أفهم أن مشاركتي في هذا المشروع تتضمن مهمة تقديم أفكاري ومشاعري حول FertiSTAT أثناءالأداة. سيتم بعد ذلك إكمال مقابلة قصيرة واستبيان عن معلوماتي الديموغرافية (على سبيل المثال، العمر، والتعليم). سوف تأخذ الدراسة بأكملها حوالي 60 إلى 90 دقيقة للإكمال.

وأنا أفهم أن مشاركتي في هذه الدراسة طوعية تماما وأستطيع الانسحاب من الدراسة في أي وقت دون إبداء أسباب وأنا حر أن أسأل أي سؤال في أي وقت. أنا حر في الانسحاب أو مناقشة مخاوفي مع الباحثة رشا بيومي ([bayoumir@cardiff.ac.uk](mailto:bayoumir@cardiff.ac.uk)).

أنا أفهم أن المقابلة ستكون مسجلة حتى يمكن نسخها وتوليفها. سيتم تخزين المعلومات التي قدمتها على جهاز كمبيوتر (محمي بكلمة مرور) المملوك لدى الباحثة رشا بيومي والأستاذ جاكي بوافين وسيعقد مجهول، بحيث أنه من المستحيل أن تتبع هذه المعلومات لي على حدة. وبمجرد أن يتم نسخ تسجيل المقابلة سيتم حذف التسجيل وسيتم الاحتفاظ بالبيانات والردود على الاستبيان إلى أجل غير مسمى.

أفهم أيضا أنه في نهاية الدراسة ستقدم لي معلومات حول الغرض من الدراسة.

أنا، ___________________________________ (الاسم) موافق على المشاركة في الدراسة التي أجرتها رشا بيومي (كلية علم النفس، جامعة كارديف مع إشراف البروفيسور جاكي بوافين).

التوقيع:

التاريخ:

1. **The 16-item background information form for interviews**
2. Patient number ______________
3. Age ______________
4. Sex ______________
5. Address ______________
6. Occupation ______________
7. Education:
   - Illiterate:
   - Primary level:
   - Secondary level:
   - More than secondary level:
8. Duration of marriage ______________
9. Duration of couple living together ______________
10. Menstruation:
    - Normal
    - Not normal
11. If menstruation is not normal:
    - No period >6 months
    - No period <6 months
    - Increase in the amount of menstruation
    - Decrease in the amount of menstruation
12. Painful intercourse
    - Yes
    - No
13. Medical and surgical history:
    - Blood pressure (hypertension)
    - Thyroid disease
14. Previous pregnancy
    - Yes
    - No
15. Number of previous pregnancies ______________
    - Without treatment ______________
    - With ovarian stimulation only ______________
    - With ART ______________
16. Duration of delay in pregnancy ______________
17. Reasons for delay in pregnancy:
    - Husband
    - Wife
    - Both
    - Unknown
18. **Semi-structured interview topic guide**

| **Question** | **Section** |
| --- | --- |
|  | **Section 1: Fertility knowledge**  (before administering FertiSTAT) |
| 1 | Do you know about the signs and symptoms of infertility? |
| 2 | What are some signs and symptoms that you know? (if not understood, explain) |
| 3 | Do you have information about the risk factors that people can avoid? (if not understood, explain) |
| 4 | What are some risk factors that you know? |
| 5 | Do you know when a person should consult a doctor for delayed pregnancy? |
| 6 | Would you like to know more about the signs, symptoms, preventable risk factors and when to seek help? |
|  | **Section 2: Questions about FertiSTAT**  (after administering FertiSTAT) |
|  | 1. **Benefit of FertiSTAT** |
| 7 | Were you aware of this information before? |
| 8 | What information is new to you? |
| 9 | Would you have wanted to know this information in the past? |
| 10 | Do you think this information is important for people to know, here in Sudan, or is it unrelated to our society? |
| 11 | How useful would it have been to have this information and when? |
| 12 | Is this information beneficial? |
| 13 | In what way is this information beneficial? |
|  | 1. **Format, setting, source and target population**   (if unable to generate spontaneously, give examples) |
| 14 | Where can people get this information from? What is the best setting? |
| 15 | Who is the best person to convey this type of information? |
| 16 | How can this information be conveyed? |
| 17 | What if you find it in a magazine or a newspaper, would that be acceptable? |
| 18 | When is the best timing to present this information? |
| 19 | What age is this information most appropriate for? |
| 20 | Should the information be given before or after marriage? |
|  | 1. **Sensitive topics in FertiSTAT** |
| 21 | We have been told that some of the topics in FertiSTAT may not be acceptable in our society, that there are things we shouldn’t say, what do you think? |
| 22 | What about information about things like drugs, alcohol and sex, how acceptable would it be to talk about them in our society? |
| 23 | Is it better to talk about these sensitive topics or to avoid them? |
| 24 | What would be the best way to talk about these topics? |

1. **Debrief for interviews**

One of the most important issues in determining health is how we perceive our own health and illness. Successful public health campaigns have used a strategy of increasing public awareness of certain illnesses by researching the relevant health indicators for each illness, ensuring most people are aware of the signs and symptoms of the diseases (e.g. cancer, heart disease). Such research has highlighted that this can be used to monitor needs for health care, and evaluate the effectiveness and impact of healthcare programmes.

The majority of couples will get pregnant after trying for 12 months. However, for a small number of couples, it may take longer. There has been little research highlighting the main indicators for those that might take longer to get pregnant. In addition, few people know the signs of reproductive disease or the risk factors for fertility difficulties. A tool was developed to raise awareness about risk factors for fertility problems and provide women with information on what to do when they have any risks. We asked you to give us your thoughts and feelings about FertiSTAT, and answer questions in an interview and questionnaire to enable us to evaluate whether these could be used at this and other clinics in Sudan.

It was important to ask a range of personal questions about your lifestyle and reproductive history, and we would like to assure you that all the data you provided will be held anonymously and it will not be possible to trace the information back to you. Data will be stored on a computer that is password-protected and belongs to Rasha Bayoumi and Prof. Jacky Boivin.

If participation in the study has caused concern about your health, please contact your doctor in the usual way or this Facebook page – OBGYN consultations, that provides support to women with fertility problems.

If you have any further questions about this research, please let Rasha Bayoumi or your doctor know of these concerns and they will inform Prof. Jacky Boivin.

School of Psychology

Cardiff University

Tower Building, Park Place

Cardiff, Wales

CF10 3AT

boivin@cardiff.ac.uk

Prof. Jacky Boivin is interested in the psychosocial aspects of reproductive health. She has conducted many studies in this area on issues such as the link between stress and fertility, differences between men and women in emotional reactions to fertility issues, whether counselling helps people cope with fertility problems, how children conceived with fertility treatment develop, and much more.

This research has been carried out with the help of women from many countries worldwide. You can see some of the published reports of this work on [www.cardifffertilitystudies.com](http://www.cardifffertilitystudies.com).

**Psychology ethics committee details:**

Email:  [psychethics@cf.ac.uk](mailto:psychethics@cf.ac.uk)

Phone: +44 (0)29 208 74007, Fax: +44 (0)29 2087 4858. Address: Psychology Ethics Committee Secretary.

**Arabic Debrief**

استجوب (شرح الغرض من المقابلة والاستبيانات)

واحدة من أهم القضايا في تحديد الصحة هي الطريقة التي ننظر بها لصحتنا و الأمراض. وقد استخدمت حملات الصحة العامة الناجحة استراتيجية لزيادة الوعي عن بعض الأمراض عن طريق البحث في المؤشرات الصحية المناسبة لكل مرض، وضمان أن معظم الناس يدركون علامات وأعراض الأمراض (مثل السرطان وأمراض القلب). وقد أبرزت هذه البحوث أن هذا النهج يمكن استخدامه لرصد احتياجات الرعاية الصحية، وتقييم مدى فعالية وتأثير برامج الرعاية الصحية.

بالنسبة لغالبية الأزواج يحدث الحمل بعد المحاولة لمدة اثني عشر شهرا. ومع ذلك، لعدد من الأزواج قد يستغرق وقتا أطول. هناك القليل من الأبحاث عن المؤشرات الرئيسية لتأخر الحمل. وبالإضافة إلى ذلك قلة من الناس تعرف علامات المرض التناسلي أو عوامل صعوبات الخصوبة. وقد تم تطوير أداة لرفع مستوى الوعي حول عوامل الخطر لمشاكل الخصوبة وتزويد النساء بالمعلومات حول ما يجب القيام به عندما يكون لديهم أي مخاطر. ومع ذلك، فإننا لا نعرف ما إذا كانت هذه الأدوات يمكن أن تستخدم في بلدان غير حيث تم وضعها.

طلبنا منك أن تعطينا أفكارك ومشاعرك حول FertiSTAT والإجابة على الأسئلة في المقابلة والاستبيان لنتمكن من تقييم ما إذا كانت هذه الاستبيانات يمكن استخدامها في هذه العيادة وغيرها من العيادات في السودان.

كان من المهم طرح مجموعة من الأسئلة الشخصية حول نمط حياتك وتاريخ الإنجاب، ونود أن نؤكد لكم أن جميع البيانات التي قدمتها ستعقد مجهول، وأنه لن يكون من الممكن تتبع المعلومات مرة أخرى لك. سيتم تخزين البيانات على جهاز كمبيوتر (محمي بكلمة مرور) وينتمي إلى/المملوك لدى رشا بيومي والأستاذ جاكي بوافين.

إذا كان الاشتراك في هذه الدراسة قد تسبب في القلق بشأن صحتك الرجاء الاتصال بالطبيب بالطريقة المعتادة أو عن طريق صفحة الفيسبوك – استشارات أمراض النساء والتوليد و الخصوبة، التي توفر الدعم للنساء الذين يعانون من مشاكل الخصوبة.

إذا كان لديك أي أسئلة أخرى حول هذا البحث اسأل رشا بيومي أو طبيبك عن هذه المخاوف، وأنهم سوف إبلاغ الأستاذ جاكي بوافين.

كلية علم النفس

جامعة كارديف

برج البناء، بارك بليس

كارديف، ويلز

CF10 3AT

boivin@cardiff.ac.uk

أستاذة جاكي بوافين تهتم بالجوانب النفسية والاجتماعية للصحة الإنجابية. وقالت إنها أجرت العديد من الدراسات في هذا المجال على قضايا مثل العلاقة بين التوتر والخصوبة، والاختلافات بين الرجال والنساء في ردود الفعل العاطفية لقضايا الخصوبة، إذا الإرشاد يساعد الناس على التكيف مع مشاكل الخصوبة، نمواطفال الانابيب، والكثير.

وقد تم تنفيذ هذا البحث مع مساعدة نساء من العديد من البلدان في جميع أنحاء العالم. يمكنك ان ترى بعض التقارير التي نشرت على www.cardifffertilitystudies.com.

تفاصيل لجنة الأخلاق بكلية علم النفس:

البريد الإلكتروني: psychethics@cf.ac.uk

الهاتف: +44 (0) 29 208 74007 فاكس: +44 (0) 29 2087 4858. العنوان: أمين سر لجنة الأخلاق بكلية علم النفس.
